# Increased betulinic acid induced cytotoxicity and radiosensitivity in glioma cells under hypoxic conditions

**DOI:** 10.1186/1748-717X-6-111

**Published:** 2011-09-09

**Authors:** Matthias Bache, Martin P Zschornak, Sarina Passin, Jacqueline Keßler, Henri Wichmann, Matthias Kappler, Reinhard Paschke, Goran N Kaluđerović, Harish Kommera, Helge Taubert, Dirk Vordermark

**Affiliations:** 1 Department of Radiotherapy, Martin-Luther-University Halle-Wittenberg, Dryanderstr. 4, 06110 Halle, Germany; 2 Department of Oral and Maxillofacial Plastic Surgery, Martin-Luther-University Halle-Wittenberg, Ernst-Grube-Str. 40, 06120 Halle, Germany; 3 Biozentrum, Martin-Luther-Universität Halle-Wittenberg, Weinbergweg 22, 06120 Halle, Germany; 4 Div. Molecular Urology, Clinic of Urology, University Hospital Erlangen, Erlangen, Germany and Nikolaus-Fiebiger-Center for Molecular Medicine, Friedrich Alexander University Erlangen-Nürnberg, Germany

**Keywords:** betulinc acid, glioma cells, hypoxia, irradiation

## Abstract

**Background:**

Betulinic acid (BA) is a novel antineoplastic agent under evaluation for tumor therapy. Because of the selective cytotoxic effects of BA in tumor cells (including gliomas), the combination of this agent with conservative therapies (such as radiotherapy and chemotherapy) may be useful. Previously, the combination of BA with irradiation under hypoxic conditions had never been studied.

**Methods:**

In this study, the effects of 3 to 30 μM BA on cytotoxicity, migration, the protein expression of PARP, survivin and HIF-1α, as well as radiosensitivity under normoxic and hypoxic conditions were analyzed in the human malignant glioma cell lines U251MG and U343MG. Cytotoxicity and radiosensitivity were analyzed with clonogenic survival assays, migration was analyzed with Boyden chamber assays (or scratch assays) and protein expression was examined with Western blot analyses.

**Results:**

Under normoxic conditions, a half maximal inhibitory concentration (IC_50_) of 23 μM was observed in U251MG cells and 24 μM was observed in U343MG cells. Under hypoxic conditions, 10 μM or 15 μM of BA showed a significantly increased cytotoxicity in U251MG cells (p = 0.004 and p = 0.01, respectively) and U343MG cells (p < 0.05 and p = 0.01, respectively). The combination of BA with radiotherapy resulted in an additive effect in the U343MG cell line under normoxic and hypoxic conditions. Weak radiation enhancement was observed in U251MG cell line after treatment with BA under normoxic conditions. Furthermore, under hypoxic conditions, the incubation with BA resulted in increased radiation enhancement. The enhancement factor, at an irradiation dose of 15 Gy after treatment with 10 or 15 μM BA, was 2.20 (p = 0.02) and 4.50 (p = 0.03), respectively. Incubation with BA led to decreased cell migration, cleavage of PARP and decreased expression levels of survivin in both cell lines. Additionally, BA treatment resulted in a reduction of HIF-1α protein under hypoxic conditions.

**Conclusion:**

Our results suggest that BA is capable of improving the effects of tumor therapy in human malignant glioma cells, particularly under hypoxic conditions. Further investigations are necessary to characterize its potential as a radiosensitizer.

## Background

Glioblastoma is the most frequent primary brain tumor and is characterized by a poor patient prognosis. Although radiotherapy is widely used for the treatment of patients with glioblastoma, the intrinsic radioresistance of these tumors remains a critical problem in the management of such patients [1]. Betulinic acid (BA) represents a new therapeutic agent with possible use in the treatment of glioblastoma. BA, a pentacyclic triterpene, can be synthesized by the oxidation of betulin, a substance found in the bark of birch trees. Additionally, it can also be directly isolated from certain plants. BA has several therapeutic uses. It has been used to treat inflammation, malaria, HIV and as an antimicrobial drug. In addition, BA seems capable of improving tumor therapies. For example, BA is cytotoxic in different tumor cell lines, including neuroectodermal tumors, melanoma, colon, lung and ovarian carcinoma, head and neck cancers and sarcoma [2-4]. Experiments in animals revealed that BA also has an antitumor effect in vivo [5-7]. Interestingly, the cytotoxicity of BA in tumor cells occurs regardless of whether there is a genetic defect in p53 [6,8]. Remarkably, untransformed, normal cells (in comparison to tumor cells) seem to tolerate relatively high concentrations of BA. Thus, BA is not toxic up to a concentration of 200-400 mg/kg of body weight in rats or 500 mg/kg of body weight in mice [5,9].

Different studies have shown that BA induces apoptosis [8,10-13]. In addition, BA's effects on cell migration, cell invasion and angiogenesis inhibition have been demonstrated [14-16]. Furthermore, reactive oxygen radicals generated by BA have been shown to cause significant DNA damage [17-19]. The finding that BA can both induce the formation of reactive oxygen radicals and induce apoptosis could make it attractive for the treatment of hypoxic tumors. The role of hypoxia in developing a more aggressive tumor phenotype in glioma has been previously discussed [20-22]. Because of the selective and wide-range cytotoxic effects of BA in tumor cells, the combination of BA with conservative therapies (such as radiotherapy and chemotherapy) seemed like a promising therapeutic strategy to investigate. Indeed, investigations have shown that BA enhances the cytotoxic effects of vincristine in the B16F10 melanoma cell line [7]. Additionally, sublines of SNU-C5 colon cancer cells that are resistant to chemotherapy showed a significantly increased cytotoxicity when either 5-fluorouracil, irinotecan, or oxaliplatin were combined with BA treatment [23]. Two studies examining the combination of BA and radiotherapy (in melanoma or head and neck cancer cell lines) detected an additive effect on clonogenic survival [24,25]. However, there have been no studies examining BA treatment in combination with irradiation under hypoxic conditions. In this study, we analyzed the effects of BA on the cytotoxicity, migration, protein expression of PARP, survivin and HIF-1α and radiosensitivity under normoxic and hypoxic conditions in the radioresistant glioma U251MG and U343MG cell lines.

## Methods

### Cell culture conditions, treatments with BA and irradiation

The human malignant glioma cell lines U251MG and U343MG (American Type Culture Collection) were grown in RPMI 1640 medium (Lonza, Walkersville, MD, USA) containing 10% fetal bovine serum (PAA, Cölbe, Germany), 1% sodium pyruvate (Invitrogen, Karlsruhe, Germany), 185 U/ml penicillin (Invitrogen) and 185 μg/ml streptomycin (Invitrogen) at 37°C in a humidified atmosphere containing 3% CO_2_. Hypoxia (< 1% O_2_) was achieved using a gas generator system as previously described [26]. All experiments were performed with cells in their logarithmic growth phase. BA (Biosolution GmbH, Halle, Germany) was dissolved in dimethyl sulfoxide (DMSO) to achieve a 20 mM stock solution. Cells (3 × 10^5^) were seeded in 25 cm^2 ^flasks 24 h before treatment with 3 to 30 μM BA. Cells were treated with BA or DMSO for 24 h at 37°C under normoxic or hypoxic conditions. Additionally, cells were irradiated in tissue culture flasks (Greiner, Frickenhausen, Germany) with 2, 6 or 15 Gy 24 h after incubation with BA. Irradiation was accomplished with 6 MV photons and adequate bolus material on a SIEMENS ONCOR (Erlangen, Germany) linear accelerator at a dose rate of 2 Gy/min. At 1, 24 or 48 h after irradiation, cells were harvested for clonogenic assays, protein extraction and migration assays.

### Clonogenic survival assays and radiosensitivity

The cytotoxicity of BA was evaluated using the clonogenic survival assay. The cells were trypsinized 1 h after irradiation. Based on the optimal plating efficacy (depending on the BA treatment and irradiation dose), 500-5,000 cells were seeded in 25 cm^2 ^flasks. The cells were cultured in RPMI supplemented with 10% FCS in a humidified atmosphere of 3% CO_2 _at 37°C. The medium was changed after 5 days. Between 10 and 14 days after irradiation, the cells were fixed with paraformaldehyde (Sigma, Deisenhofen, Germany), and colony formation was visualized by staining with 10% Giemsa solution (Sigma, Deisenhofen, Germany). Only colonies with > 50 cells were scored to determine the surviving fraction (SF). The cytotoxicity of BA was defined as the ratio of colonies formed after treatment with different concentrations of BA to DMSO-treated control cells. The SF was defined as the ratio of colonies formed after irradiation with 0, 2, 6 or 15 Gy to the number of colonies formed in the unirradiated controls. The enhancement factor (EF) was defined as the ratio of the SF of BA-treated cells to DMSO-treated control cells dependent on the dose of irradiation. The data represent at least three independent experiments.

### Migration assays and cell cycle analysis

Cell migration was assessed using modified Boyden chambers as previously described [27]. Cells (2 × 10^4^) were suspended in 300 μl of RPMI without FCS and were then added to the upper chamber (membrane filter with 8 μm pore size), while the bottom chamber was filled with 1 ml of RPMI supplemented with 20% FCS (as a chemoattractant). The assay was performed at 37°C in a humidified atmosphere containing 3% CO_2 _for at least 16 h. Non-migrating cells on the upper side of the transwell inserts were removed. The cells that had migrated to the bottom side of the membrane were trypsinized and counted with CASY DT (Schärfe System GmbH, Reutlingen, Germany). The data represent at least three independent experiments.

Furthermore, we used a wound scratch assay to determine the migration of cells after treatment with BA. Cells were grown in 6-well cell culture plates in RPMI medium containing 10% FCS and were cultured to 100% confluence. A uniform cell-free area was created by scratching the confluent monolayer with a 200 μl pipette tip. To determine the migration of glioma cells, the wound closure was observed at different time points. The wound scratch assay was performed three times in independent experiments.

Cells were analyzed for cell cycle distribution. About 5 × 10^5 ^cells were harvested and washed in PBS. Subsequently, 95% ethanol was added slowly until a final concentration of 80% was reached. The DNA content, which was indicated by the extent of staining of propidium iodide, was measured by flow cytometry in an FACSscan (Becton Dickinson, Heidelberg, Germany), using the CellFit software (Version 2.0).

### Western blotting

Cells were washed, trypsinized and centrifuged. The supernatant of cells was washed with PBS and resuspended in 100 μl of lysis buffer (50 mM Tris at pH 8.0, 0.3 M NaCl, 1 mM EDTA, 0.5 mM dithiothreitol, 0.1% NP40 and protease inhibitors), followed by ultrasonic homogenization. After centrifugation at 14,000 g for 15 min, the supernatant was collected and the protein concentration was determined using the Bradford assay (BioRad, Munich, Germany). About 30 μg of total protein from each cell lysate was separated on a 10% NuPAGE Bis-Tris (Invitrogen) gel that was placed in an X-Cell SureLock Mini-Cell (Invitrogen). The membrane was blocked with 10% non-fat milk in TBST (50 mM NaCl, 30 mM Tris-HCl at pH 8.0 and 0.1% Tween) for 1 h and incubated with rabbit anti-human survivin antibody (1:1,000 dilution, clone AF886, R&D Systems, Wiesbaden, Germany), rabbit anti-human cleaved PARP (1:2,000, Cell Signaling, Danvers, MA, USA), mouse anti-human HIF1α antibody (1:1,000, BD Transduction Laboratories, Lexington, KY) and mouse anti-β-actin (1:5,000, Sigma, Deisenhofen, Germany) at 4°C overnight. After washing, the membranes were incubated with a horseradish peroxidase-labeled goat anti-rabbit or anti-mouse IgG (1:2,000, DAKO, Glostrup, Denmark) for 1 h at room temperature. For protein detection, membranes were incubated with ECL substrate or ECL Plus Blotting Detection System for 1 min (Amersham Pharmacia Biotech, Freiburg, Germany) and exposed to X-ray film (Biomax, Kodak, Braunschweig, Germany).

### Statistical analyses

The experimental results were analyzed by paired Student's t-tests. A p-value of 0.05 was considered to be significant.

## Results

### Effects of BA on clonogenic survival

**Figure 1:**
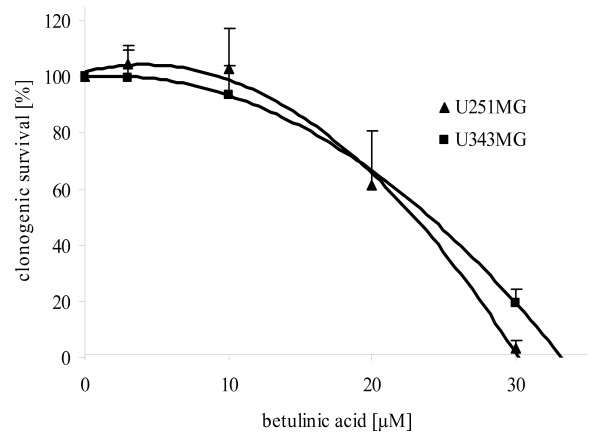
**Cytotoxicity in U251MG and U343MG cell lines after treatment with BA**. Both glioma cell lines were treated with increasing doses of BA from 3 - 30 μM. The half maximal inhibitory concentration (IC_50_) with an incubation time of 24 h was 24 μM in U343MG cells and 23 μM in U251MG cells. Data represent mean values (± SD) of three independent experiments.

**Figure 2:**
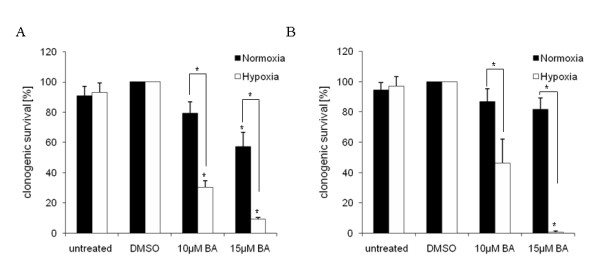
**Effects of BA on clonogenic survival of glioma cells under normoxic or hypoxic conditions**. Clonogenic survival in U251MG (A) and U343MG (B) cells after treatment with 10 or 15 μM BA under normoxic or hypoxic conditions. Under hypoxia, when compared to normoxic conditions, BA showed increased cytotoxicity in both glioma cell lines. Data represent mean values (± SD) of three independent experiments (* p < 0.05).

The effects of BA on the clonogenic survival, cell migration, cell cycle, protein expression and radiosensitivity in U251MG and U343MG glioma cell lines under normoxic and hypoxic conditions were determined. With higher concentrations (from 3 - 30 μM), a decline in clonogenic survival was observed, with an IC_50 _of 23 μM in U251MG cells and 24 μM in U343MG cells under normoxic conditions after an incubation time of 24 h (Figure 1). In addition, longer incubation with BA led to increased cytotoxicity in both cell lines (data not shown). Additionally, incubation of BA caused an increase of subG1-cells in both cell lines. However, we did not find effects of BA on cell distribution in other cell cycle phase (data not shown). Under hypoxic conditions, BA had significantly increased cytotoxicity in both glioma cell lines (Figure 2). After a 24 h incubation with 10 μM or 15 μM BA under normoxic conditions, the clonogenic survival was reduced to 79% (p = 0.07) or 57% (p = 0.03) in U251MG cells and 87% (p = 0.15) or 82% (p = 0.07) in U343MG cells, respectively. Under hypoxic conditions, an increased reduction in survival to 30% (p = 0.01) or 9% (p = 0.03) and 46% (p = 0.10) or 0.8% (p = 0.03), respectively, was detected (Figure 2).

### Effects of BA on cell migration and protein expression

**Figure 3:**
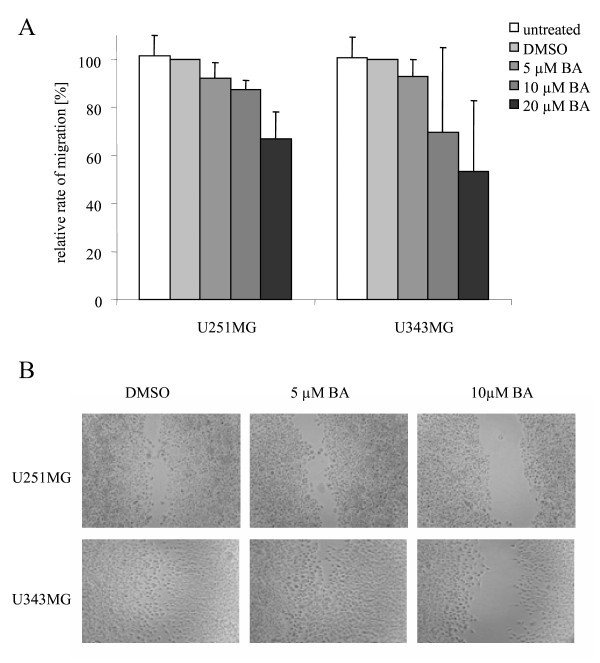
**Effects of BA on cell migration of glioma cells**. Migration rates of U251MG and U343MG cells treated with BA analyzed by Boyden chamber assays (A) and scratch assays (B) under normoxic conditions. Compared to DMSO-treated control cells, incubation with 5, 10 and 20 μM BA led to a decrease in cell migration rates in both glioma cell lines. Similarly, cells had a reduced migration rate after BA treatment as measured by the scratch assay. Data represent the average values (± SD) of three independent experiments.

The effects of BA on the migration rates of both glioma cell lines were determined with the Boyden chamber assay and the scratch assay. Decreased migration rates were detected after incubation with a higher concentration of BA in both cell lines. Compared to DMSO-treated control cells, incubation with 5, 10 and 20 μM BA led to decreased cell migration rates in U251MG cells to 92% (p = 0.21), 87% (p = 0.12) and 67% (p = 0.09), or in U343MG cells to 93% (p = 0.10), 70% (p = 0.20) and 53% (p = 0.08), respectively, under normoxic conditions (Figure 3A). Similarly, reduced migration rates were found after cells were incubated with BA in the scratch assay (Figure 3B).

**Figure 4:**
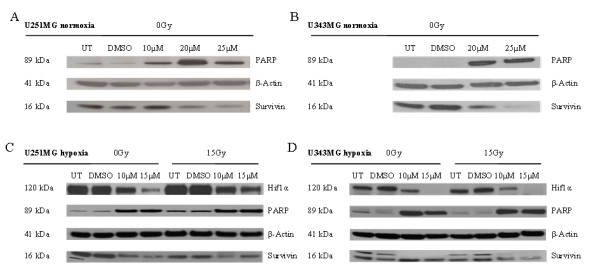
**Effects of BA and irradiation on protein expression levels of glioma cells**. BA treatment affects the cleavage of PARP, the expression of survivin and hypoxia-induced HIF-1α protein levels in U251MG (left) and U343MG (right) cells. Cell lines were untreated (UT), treated with DMSO or with increasing doses of BA from 10, 20 or 25 μM under normoxic conditions (A, B) and untreated (UT), treated with DMSO or with doses of 10 or 15 μM BA plus irradiation at 15 Gy under hypoxic conditions (C, D). Actin served as an internal loading control. The Western blot shows one representative result out of three independent experiments.

Using Western blot analysis, we examined the cleavage of PARP (as an indicator for the induction of apoptosis) and the expression of survivin (as an inhibitor of apoptosis) (Figure 4). Incubation with 20 or 25 μM BA led to PARP's cleavage, and to a decrease in survivin levels under normoxic conditions. Additionally, increased PARP's cleavage and a decrease in survivin protein levels were observed after treatment with 10 or 15 μM BA in the U251MG cells under hypoxic conditions. BA also decreased hypoxia-induced levels of the HIF-1α protein in both cell lines (Figure 4). However, combination of BA with radiotherapy showed no additional effects on PARP cleavage or the expression of survivin under normoxic or hypoxic conditions.

### Effects of BA on radiosensitivity

**Figure 5:**
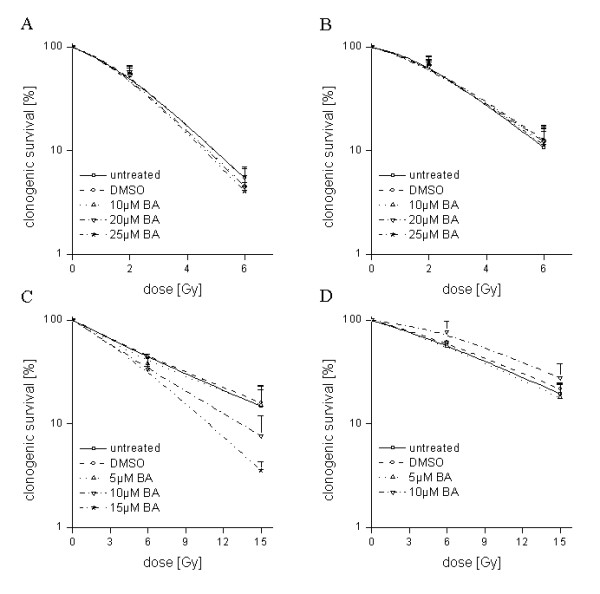
**Effects of BA on radiosensitivity of glioma cells**. U251MG (left) and U343MG (right) cells were either treated with 10, 20 or 25 μM BA and irradiated with a dose of 2 and 6 Gy under normoxic conditions (A, B), or treated with 5, 10 or 15 μM BA and irradiated with a dose of 6 and 15 Gy under hypoxic conditions (C, D) and compared to DMSO-treated control cells. Data represent the mean values (± SD) of three independent experiments.

Irradiation at 2 Gy reduced clonogenic survival to 70% (SF2 = 0.70) in U251MG cells and 71% (SF2 = 0.71) in U343MG cells under normoxic conditions. Irradiation-induced clonogenic survival of U251MG and U343MG cells was increased under hypoxic conditions when compared to normoxic conditions (Figure 5). The combination of BA with radiotherapy resulted in an additive effect for U343MG cells under normoxic and hypoxic conditions. However, a weak not significant radioprotective effect was observed at 10 μM BA under hypoxic conditions. In addition, a weak radiation enhancement was observed for U251MG cells under normoxic conditions. The enhancement factor at a radiation dose of 6 Gy after treatment with 20 μM and 25 μM BA was 1.22 (p = 0.02) and 1.34 (p = 0.15), respectively (Figure 5). However, under hypoxic conditions, the effects of BA on radiosensitivity were strongly enhanced in U251MG cells. The enhancement factor at an irradiation dose of 15 Gy after 10 μM or 15 μM BA treatment was 2.20 (p = 0.02) and 4.50 (p = 0.03), respectively (Figure 5).

## Discussion

Betulinic acid (BA) represents a new therapeutic agent with possible uses in the treatment of glioblastoma [10]. Because of the selective cytotoxic effects of BA in tumor cells, combining BA treatment with conservative tumor therapies (such as radiotherapy and chemotherapy) is attractive. Here, we report that BA triggers cytotoxicity in human malignant glioma cells in a dose-dependent manner (Figure 1). In addition, the cytotoxic effects of BA were increased in both cell lines under hypoxic conditions (Figure 2). In accordance with our investigations, BA was found to be a highly potent cell-death promoting agent in primary glioblastoma cells and cell lines [10,28]. However, 17% (4 of 24) primary glioblastoma cells did not respond to treatment with BA [10]. An activated EGFR/AKT pathway and the expression of survivin contributed to a lower sensitivity in response to BA treatment in human melanoma cells [29].

In the present study, the increased cytotoxicity in both glioma cell lines was dependent on BA concentration. Additionally, it was coupled with an inhibition of cell migration, the cleavage of the apoptotic protein PARP and a decrease in the protein level of the apoptosis inhibitor survivin (Figure 3 and 4). In agreement with our current findings, BA was also found to inhibit the migration of glioma (C6), lung carcinoma (A549) and medulloblastoma (TE671) cells [15]. In addition, BA-induced inhibition of migration was associated with the suppression of mRNA and protein levels of MMP-2 and MMP-9 in vascular smooth muscle cells [30]. It is well known that the activation of these two matrix metalloproteinases is involved in cellular invasion and migration. Recent studies also detected BA as an inhibitor of migration, invasion and angiogenesis [14,16]. Furthermore, different analyses have shown that BA induces apoptosis in tumor cell lines [8,11,12,31]. BA-induced apoptosis can be associated with cytochrome c release, the activation of caspases, the cleavage of PARP and modulation of Bcl2 family protein levels in glioma cells [10,17,32]. However, overexpression of the anti-apoptotic protein Bcl-2 only partially delayed the induction of apoptosis in Jurkat cells [13]. Somewhat controversial is the finding that in head and neck cancer cells, BA-induced cytotoxic effects were linked to a decreased level of Bax, an inducer of apoptosis [33]. In prostate cancer cells, the combination of docetaxel and BA increased NF- κB activity and stimulated apoptosis [34]. Altogether, the exact mechanisms by which BA might act as an effective and wide-range anti-cancer agent remain unclear.

First investigations studying the effect of BA treatment in combination with other chemotherapeutic drugs showed that BA improved the cytotoxic effects of different agents. In the mouse melanoma cell line B16F10, BA improved vincristine-induced cytotoxic effects in vitro, in addition to reducing the number of metastases in vivo [7]. Sublines of the colon cancer cell line SNU-C5 that are resistant to chemotherapy showed significantly increased cytotoxicity when 5-fluorouracil, irinotecan or oxaliplatin were combined with BA treatment [23]. In addition, BA augmented doxorubicin- or cisplatin-induced apoptosis in several different tumor cell lines, while no apoptosis was induced by BA treatment in untransformed fibroblasts [31]. However, the addition of BA in SCC9 and SCC25 head and neck tumor cell lines had no effects on cisplatin-induced apoptosis [35]. In recent studies, glioma cell lines were characterized as radioresistant, with a low rate of irradiation-induced apoptosis [36-38]. Our analyses show that BA, in combination with radiotherapy, resulted in an additive effect for the U343MG cells and a weak radiation enhancement for U251MG cells under normoxic conditions (Figure 5). This is in agreement with two studies that dealt with testing a combination of BA treatment and radiotherapy for its effects on two melanoma [24] and two head and neck cancer cell lines [25]. These studies showed that these two treatments were more effective in combination. The present data also demonstrate that BA strongly enhances the radiosensitivity of U251MG cells under hypoxic conditions (Figure 5). To our knowledge, this is the first study demonstrating that BA can increase cytotoxicity and radiosensitivity under hypoxic conditions. These effects are coupled with the inhibition of the hypoxia-induced increase of HIF-1α protein level (Figure 4). In accordance with results presented here, a decrease of HIF-1α after BA treatment has been described in adenocarcinoma cells [39]. Recently, our group showed that the silencing of HIF-1α by siRNA or chetomin resulted in a significantly enhanced cytotoxicity and radiosensitivity in both human glioma cell lines [38], in addition to HT1080 human fibrosarcoma cells [40,41]. The downregulation of HIF-1α consistently increased the sensitivity of human glioma cells to doxorubicin and etoposide [42].

## Conclusions

In summary, BA affects the clonogenic survival, migration and apoptosis in human malignant glioma cells. Remarkably, additional effects on cytotoxicity and radiation sensitivity were observed under hypoxic conditions. These results suggest that BA may be suitable for improving the treatment of malignant gliomas. However, more investigations are necessary to characterize its role as chemotherapeutic drug and potential radiosensitizer.

## List of abbreviations

BA: betulinic acid, IC_50_: half maximal inhibitory concentration, SF: survival fraction, EF: Enhancement factor, UT: untreated, DMSO: dimethyl sulfoxide

## Competing interests

The authors declare that they have no competing interests.

## Authors' contributions

MB and DV designed the study, analyzed the data and drafted the manuscript.

MPZ, SP performed experimental procedures, analyzed the data and drafted the manuscript.

JK, HW, MK, RP, GNK, HK and HT aided in study design, analyzed the data and reviewed the manuscript. All authors read and approved the final manuscript.
